# A Rare Complication of Phenytoin Infusion in Newborn: Purple Glove Syndrome

**DOI:** 10.4274/balkanmedj.2017.0480

**Published:** 2017-12-01

**Authors:** Dilek Ulubaş Işık, Nihal Demirel, Sara Erol, Sezin Ünal, Ahmet Yağmur Baş

**Affiliations:** 1 Clinic of Neonatology, Etlik Zübeyde Hanım Women’s Health Training and Research Hospital, Ankara, Turkey

A 2190 g male neonate was born at 34 weeks of gestation by emergency cesarean section (fetal bradycardia). He required resuscitation after birth and admitted to the intensive care unit due to prematurity and perinatal asphyxia. The cranial ultrasonography excluded intraventricular hemorrhage and other significant abnormalities. Sepsis was ruled out regarding sterile blood cultures and subsequent negative serum C reactive protein levels. He had tonic-clonic convulsions in the upper extremities on the second day of life refractory to ongoing phenobarbital, midazolam treatments, thus phenytoin infusion was prescribed. Phenytoin was diluted with saline solution to achieve final concentration of 5 mg phenytoin per mL. There existed no visible precipitants. After the peripheral line was controlled for venous patency, drug infusion was initiated. Phenytoin of 40 mg was intended to infuse over 30 minutes, through a peripheral venous cannula on the right hand. The purple-blue discoloration of the right hand and arm was observed at the 15^th^ minute of infusion. The peripheral venous cannula was removed. The right arm had become edematous in hours. Limb movements were limited. Peripheral pulses were intact. Doppler ultrasonography excluded thrombosis or occlusion. A diagnosis of Purple Glove syndrome was made. The affected limb was elevated, and heat and local nitroglycerin were applied. Multiple necrotic lesions with ill-defined margins were formed and perfusion of that limb was disturbed after 24 hrs (Figure 1). Heparin infusion for anticoagulation was given for 3 days and followed by low molecular weight heparin for 2 months. Edema and purple-blue discoloration were diminished in weeks. Besides the reduced limb movements and reflexes of the affected limb, muscle atrophy was also present at discharge, on the 40^th^ day. The written informed consent was obtained from the parents.

Purple Glove syndrome is a rare complication of intravenous phenytoin infusion. The incidence ranges from 1.7% to 5.9% Purple Glove syndrome can occur with or without clinically apparent extravasation. Highly alkaline phenytoin solution may induce vasoconstriction, which results the purple-blue discoloration, edema and tenderness of skin adjacent to the route of venous infusion (1,2). The suggested mechanisms are damage to vascular endothelial integrity due to vasoconstriction and occlusion due to phenytoin precipitates. Patients receiving phenytoin infusion faster than 25 mg/min are associated with an increased risk of Purple Glove syndrome. The clinical symptoms include pain, edema, and purple-blue discoloration of skin tissue adjacent to the site of intravenous phenytoin infusion. The symptoms usually resolve (within days to weeks), with the discoloration receding towards the intravenous site. The treatment includes pain management, elevation of the affected limb, gentle heat compression, massage, and local application of nitroglycerin and brachial plexus block (3,4). Rarely, Purple Glove syndrome may progress to necrosis, ischemia, vascular compression, or compartment syndrome and may require systemic anticoagulant therapy and surgical interventions including fasciotomy or amputation. The purpose of this report is to create awareness of this condition in neonates receiving phenytoin infusion among neonatologists.

## Figures and Tables

**FIG. 1.**
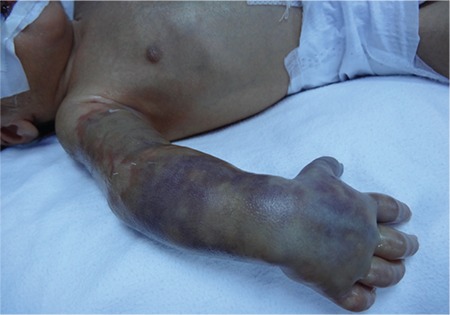
The purple-blue discoloration and edema of the right hand and arm.
